# Assessment of Temporal Selection Bias in Genomic Testing in a Cohort of Patients With Cancer

**DOI:** 10.1001/jamanetworkopen.2020.6976

**Published:** 2020-06-08

**Authors:** Kenneth L. Kehl, Deborah Schrag, Michael J. Hassett, Hajime Uno

**Affiliations:** 1 Division of Population Sciences, Dana-Farber Cancer Institute/Harvard Medical Center, Boston, Massachusetts

## Abstract

This cohort study assesses for temporal selection bias in patients with lung, breast, colorectal, pancreatic, or urothelial cancer from a single institution who had tumor profiling using a next-generation sequencing protocol between 2013 and 2017.

## Introduction

Molecular tumor profiling is now routinely performed in cancer care and research. At scale, linked tumor profiles and clinical outcomes could enable researchers to develop rich predictors of treatment effectiveness. However, profiling may be ordered in clinical practice specifically to inform treatment for worsening cancer, introducing selection bias^1^ into secondary analysis of genomic data. This would manifest not only as systematic differences between patients who entered a cohort and those who did not, but also as differences by timing of cohort entry. This analysis was conducted to evaluate such temporal selection bias in a large clinicogenomic data set.

## Methods

A retrospective single-institution cohort study was conducted of patients with lung, breast, colorectal, pancreatic, or urothelial cancer (all stages) who had tumor profiling under a next-generation sequencing protocol from July 1, 2013 to December 31, 2017.^2^ This protocol was approved by the Dana-Farber/Harvard Cancer Center Institutional Review Board; informed consent was obtained for patients who had profiling on a research basis and waived for patients who had profiling ordered as a standard clinical test. Results are presented according to the Strengthening the Reporting of Observational Studies in Epidemiology (STROBE) reporting guideline.

The association between time from diagnosis to genomic testing (left truncation time) and time from diagnosis to death was measured using the inverse probability weighted conditional Kendall τ statistic (Tc),^3,4^ which can be interpreted as a nonparametric association between left truncation and survival times. Negative values indicate that longer times to testing correlate with survival shorter than expected in the absence of dependent left truncation; positive values indicate the opposite. Hypothesis testing evaluated the null hypothesis that left truncation and survival times were quasi-independent,^3^ that is, that Tc was zero. A *P* value of .05 (2-tailed) was considered significant. Data were evaluated from October 1, 2019, to March 27, 2020.

## Results

The cohort included 4777 patients (mean [SD] age, 62.8 [12.7] years; 1767 [36.9%] men and 3010 [63.0%] women); the distribution of cancer histology included lung (2009 [42.1%]), breast (1210 [25.3%]), colorectal (823 [17.2%]), urothelial (413 [8.6%]), and pancreatic (322 [6.7%]). The overall Tc was –0.18 (95% CI, –0.21 to –0.15; *P* < .001), indicating that patients undergoing genomic testing later in their disease trajectories had survival shorter than would be expected if time to testing were independent of clinical risk. In subgroup analyses, this association was observed for patients initially diagnosed with stage I (Tc, −0.18; 95% CI, −0.32 to −0.05), stage II (Tc, −0.16; 95% CI, −0.27 to −0.06), and stage III (Tc, −0.24; 95% CI, −0.32 to −0.15) disease but not stage IV disease (Tc, 0.01; 95% CI, −0.03 to 0.06) (Figure, A), consistent with a pattern of genomic testing after relapse. The association was observed across breast (Tc, −0.27; 95% CI, −0.35 to −0.19), colorectal (Tc, −0.20; 95% CI, −0.29 to −0.11), lung (Tc, −0.17; 95% CI, −0.22 to −0.13), pancreatic (Tc, −0.10; 95% CI, −0.20 to 0.00), and urothelial (Tc, −0.27; 95% CI, −0.40 to −0.15) cancer sites (Figure, B).

**Figure.**
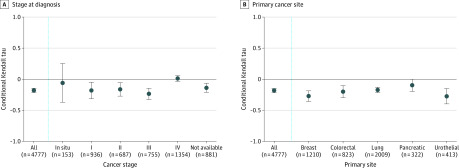
Association Between Time to Genomic Testing and Survival by Stage at Diagnosis and Primary Cancer Site A, Conditional Kendall τ (Tc) statistics measuring the association between time from cancer diagnosis to genomic testing and overall survival after diagnosis by stage at diagnosis among patients (N = 4777) with lung, breast, colorectal, pancreatic, or urothelial cancer who participated in an institutional next-generation sequencing study. B, Conditional Tc statistics measuring the association between time from cancer diagnosis to genomic testing and overall survival after diagnosis by primary cancer site. Whiskers indicate 95% CIs.

## Discussion

This analysis found an association between the timing of genomic testing and mortality risk in a clinicogenomic cohort of patients with cancer. Outcome events in such a cohort are subject not just to immortal time bias (patients who died before profiling would never have been in the cohort), but also to temporal selection bias (the timing of profiling is associated with clinical risk for outcome events). Researchers should consider these biases in genomic data analyses; studies seeking predictors of recurrence in early-stage disease may be particularly vulnerable to this problem. Limitations of this study include its single-institution design; the magnitude of bias likely varies across health systems and clinical cohorts. This analysis also did not assess for a causal relationship between genomic testing and survival.

Left truncation analyses,^5^ which consider patients at risk only after genomic testing, could eliminate immortal time bias if the timing of testing were random. However, if genomic testing is ordered because of progressive cancer, left truncation time could be undesirably associated with clinical risk, systematically excluding patients with a good prognosis at each time point. Simply restricting analyses to treatments initiated after genomic profiling may also bias a cohort toward patients considered to be high risk. Instead, researchers should measure the extent of temporal selection bias by calculating the conditional Tc for their cohort. If such bias is identified, specialized methods, including copula or transformation models,^6^ can be applied to adjust for it.
